# Relation between the Macroscopic Pattern of Elephant Ivory and Its Three-Dimensional Micro-Tubular Network

**DOI:** 10.1371/journal.pone.0166671

**Published:** 2017-01-26

**Authors:** Marie Albéric, Mason N. Dean, Aurélien Gourrier, Wolfgang Wagermaier, John W. C. Dunlop, Andreas Staude, Peter Fratzl, Ina Reiche

**Affiliations:** 1 Sorbonne Universités, Université Paris 6, Laboratoire d'Archéologie Moléculaire et Structurale, UMR 8220 CNRS – Université Pierre et Marie Curie, Paris, France; 2 Department of Biomaterials, Max-Planck-Institute of Colloids and Interfaces, Potsdam, Germany; 3 Université Grenoble Alpes, Laboratoire Interdisciplinaire de Physique (LIPHY), Grenoble, France; 4 CNRS, LIPHY, Grenoble, France; 5 European Synchrotron Radiation Facility, Grenoble, France; 6 Bundesanstalt für Materialforschung und –Prüfung (BAM), Berlin, Germany; 7 Rathgen-Forschungslabor, Staatliche Museen zu Berlin, Stiftung Preußischer Kulturbesitz, Berlin, Germany; Seoul National University College of Medicine, REPUBLIC OF KOREA

## Abstract

Macroscopic, periodic, dark and bright patterns are observed on sections of elephant tusk, in the dentin part (ivory). The motifs—also called Schreger pattern—vary depending on the orientation in the tusk: on sections perpendicular to the tusk axis, a checkerboard pattern is present whereas on sections longitudinal to it, alternating stripes are observed. This pattern has been used to identify elephant and mammoth ivory in archeological artifacts and informs on the continuous tissue growth mechanisms of tusk. However, its origin, assumed to be related to the 3D structure of empty microtubules surrounded by the ivory matrix has yet to be characterized unequivocally. Based on 2D observations of the ivory microtubules by means of a variety of imaging techniques of three different planes (transverse, longitudinal and tangential to the tusk axis), we show that the dark areas of the macroscopic pattern are due to tubules oblique to the surface whereas bright areas are related to tubules parallel to it. The different microstructures observed in the three planes as well as the 3D data obtained by SR-μCT analysis allow us to propose a 3D model of the microtubule network with helical tubules phase-shifted in the tangential direction. The phase shift is a combination of a continuous phase shift of π every 1 mm with a stepwise phase shift of π/2 every 500 μm. By using 3D modeling, we show how the 3D helical model better represents the experimental microstructure observed in 2D planes compared to previous models in the literature. This brings new information on the origin of the unique Schreger pattern of elephant ivory, crucial for better understanding how archaeological objects were processed and for opening new routes to rethink how biological materials are built.

## Introduction

Sections of the tusks of Elephantoidea (elephant, mammoth and relatives) exhibit a unique macroscopic feature, called Schreger pattern, which consists of a periodic arrangement of dark and bright areas visible to the naked eye, the source of which has puzzled scientists for decades [1–6]. Like all mammalian tusks (e.g. those of warthogs, pigs, walruses), those of Elephantoidea are enlarged front teeth, comprised predominantly of dentin (ivory), a thin outer layer of cement and an inner pulp cavity [1]. While Elephantoidea ivory is compositionally very similar to tooth dentin (hydroxyapatite-collagen based material) [7], no other species’ tooth or tusk exhibits the Schreger pattern. This raises the question of which unique structural aspects of Elephantoidea ivory are generating this feature and how? Structurally speaking, as with teeth, ivory is characterized by a network of empty tubules (~2 μm wide), which are the remaining evidence of odontoblast migration from cement to pulp during tusk growth [8]. The tubular network perforates the homogeneous ivory matrix, composed mainly of densely packed mineralized collagen fibers.

**Fig 1:**
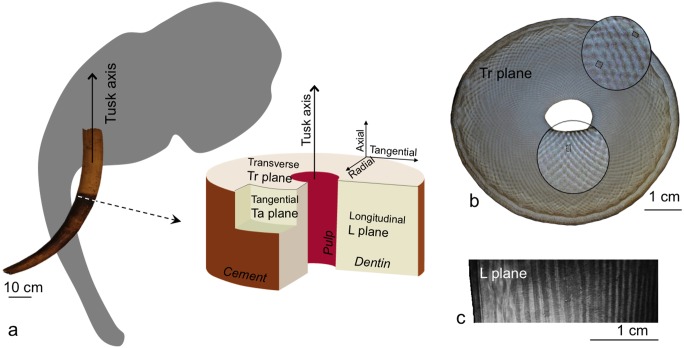
Sample orientation in the elephant tusk. (a) System of reference of elephant tusk cross-sections considered in this paper with the longitudinal plane (L plane), the tangential plane (Ta plane) and the transverse plane (Tr plane) and the respective axial, tangential and radial direction, (b) optical image of a transverse section of the tusk showing the checkerboard Schreger pattern and (c) optical image of a longitudinal section showing the alternate parallel bright and dark bands of the Schreger pattern.

The unique macroscopic pattern of Elephantoidea ivory was first reported by Daubenton in 1764 [9] and then named after Bernhard Gottlob Schreger [3]. The Schreger pattern is most striking when viewed in transverse sections (Tr plane, which is perpendicular to the tusk axis, Fig 1a), where it forms a checkerboard arrangement of bright and dark rhomboids extending in two directions, radially (from cement to pulp) and tangentially (following the cement). The shape and size of rhomboids vary across the section, from cement to pulp. For recent elephant ivory, they appear as elongated rectangles (~800 x 400 μm^2^) close to the cement, squares (~500 x 500 μm^2^) in the middle of the dentin, and elongated rectangles (~200 x 500 μm^2^) close to the pulp (Fig 1b, Figure A in S1 Fig). These changes of shape give the optical illusion of intersecting lines radiating in spiral fashion and creating the so-called Schreger angles, which vary among Elephantoidea taxa and have therefore been widely used to distinguish between mammoth and elephant ivory and even between Asiatic and African elephant ivories [10–13]. The unicity of such motifs has provided a vital diagnostic feature to fight against elephant poaching since the international ban on commercial trade in elephant ivory in 1989 [14].

In comparison with the complex checkerboard pattern of transverse sections, longitudinal sections (in line with the tusk’s longitudinal axis, L plane Fig 1a) exhibit a simpler banding pattern of alternating, parallel bright and dark bands of about 500 μm large (Fig 1c) [4]. To our knowledge, the relation between the checkerboard pattern of the transverse section and the parallel banding of the longitudinal section has never been fully described. However, this connection is of great importance, not only for a 3D understanding of what makes ivory growth unique, but also for better interpreting the pattern on manmade objects, in order to: 1) fight against illegal trade and 2) determine the location in the tusk from which carved artifacts were made, which provides information about the manufacturing processes [15].

Because of its interdisciplinary relevance, the Schreger pattern and its origin have been studied in different fields as diverse as biology, odontology, engineering material science, paleontology, archaeology, conservation and forensic sciences [1–7, 9–16]. Several authors have proposed different explanations for the histogenesis of the macroscopic Schreger pattern by considering the microstructural arrangement of elephant ivory, namely the tubular network on one hand [2–6] and the mineralized collagen fibers arrangement on the other [16].

Previous studies of the three-dimensional (3D) tubular arrangement are based on two-dimensional (2D) optical or electron microscopy observations of samples ranging from extinct elephantoids and deinotheres [6] to modern elephants [2–5]. Although authors have examined ivory in all three primary orthogonal sectioning planes—transverse, longitudinal, and tangential (Fig 1a)—their descriptions of the shape, orientation, and 3D arrangement of the dentinal tubules differ considerably. First, whereas the majority of authors stated that tubules are sinusoidal [2–4, 6], Locke proposed straight ones [5]. Second, while some authors [2–4, 6] considered the orientation of the tubule main axis to be perpendicular to the tusk axis, Locke believed tubule orientation varies from perpendicular to parallel to the tusk axis [5]. Third, Miles and White suggested tubules to be packed in groups, with all sinusoidal tubules in a group sharing the same phase, and a discrete (or stepwise) phase shift of π every 500 μm in the tangential direction, resulting in tangentially adjacent groups having opposite phases [2]. Furthermore, Virág agreed with this stepwise phase shift and additionally proposed a continuous phase shift of π every 1 mm also in the tangential direction [6]. Finally, Locke postulated that parallel straight tubules are arranged radially in longitudinal microlamina [5].

Different characteristics of the 3D tubular network have been proposed to be responsible for the emergence of the Schreger pattern: the density of tubules and their phase shift [3, 4]. Raubenheimer et al. proposed that dark bands of the longitudinal sections are due to high tubule density whereas bright ones result from low tubule density [4]. Miles and White, on the other hand, believed the stepwise phase shift of sinusoidal tubules to be the origin of the tangential staggering of bright and dark rhomboid shapes observed in transverse sections [2]. They considered their model to also account for the radial staggering of bright and dark rhomboids, with the bright and dark regions of the pattern corresponding with transverse sections of the sinusoidal tubules’ valleys and hills, respectively (see Figure B and Figure C in S1 Fig). However, from a structural viewpoint, transverse sections of radial sinusoidal tubules would produce the same 2D tubular microstructure in the Tr plane for valleys and hills (see Figure B and Figure C in S1 Fig). Therefore, if the Miles and White model is correct, then the origin of the colored staggering in the radial direction cannot be accounted by only the 3D arrangement of tubules, and other parameters need to be considered.

From this overview of the literature, we can conclude that the 3D model of the microstructure of elephant ivory still needs to be clarified and its direct relation to the macroscopic Schreger pattern determined. In this paper we attempt to unify contrasting studies on the 3D arrangement of the tubular network of elephant ivory and propose a computed model built on experimental data obtained by optical and electron microscopy observations as well as X-ray tomography analysis. We investigate the shape and the 3D arrangement of the microtubules of elephant ivory and show how this relates to the macroscopic Schreger pattern, also comparing our model with other published works by 3D modeling in order to underline which aspects of this feature still defy explanation.

## Materials and Methods

### Samples

Because of the difficulty to obtain samples and for the consistency of our study, we based our model on a single elephant tusk called MI_El_2. The tusk was provided by custom seizure the 24 June 1993 to the Director of the French Museum and housed at the *Centre de Recherche et de Restauration des Musées de France* (C2RMF), Paris. The donation was done according to the article 390 and 6 of the French custom code of the 26 September 1949 (S1 Doc). It is publicly and permanently shared between the C2RMF and the *Laboratoire d’Archéologie Moléculaire et Structurale* of the University Pierre et Marie Curie, Paris 6. The tusk, identified as African elephant, was 80 cm long and had a maximum diameter of 8 cm. One cross-section was cut in the middle of the tusk with a metal saw. The location of the cross-section in the tusk can be also evidenced by the size of the opened pulp cavity, which is quite large (1.3 cm large). At the tip of the tusk (distal part), the pulp canal is closed. This same cross-section was then sectioned into different planes. First, the cutting angle was chosen in order to obtain the longitudinal, transverse and tangential planes (Fig 1a), which are the most studied orientations considered in former papers [2–6]. Second, in order to investigate the variations in the macroscopic Schreger pattern in 3D, intermediate cutting planes were sectioned at 67°, 45° and 27°. All the results presented in this paper refer to this specific location of the cross-section in the tusk. It is known that Schreger angles vary depending on the location of the transverse section along the tusk axis [13]. Therefore, slightly different results to the ones presented here are expected in cross-sections cut in other location along the tusk axis.

### Light microscopy and scanning electron microscopy

Light microscopy (LM) was performed with a Leica (DM RXA2) microscope equipped with a DFC 480 camera on thick polished sections and thin sections of the transverse and longitudinal planes. Thick sections (200 μm) were first obtained with a diamond saw under constant water flow (Leica SP1600 saw microtome). Second, they were either polished to obtain thin sections (70 to 100 μm) or impregnated for 2 days in methylmethacrylate followed by polymerization in an oven at 50°C for 3 days, before finally being polished down to 1 μm roughness with diamond papers of increasing grade. Thin sections were studied by transmitted light whereas thick sections by reflected light.

A digital scanning electron microscope (SEM) (DSM 962, Zeiss, Oberkochen, Germany) was used for the examinations of fractured sections of the tangential plane in order to provide topographic contrast with the secondary electron mode. The fractured sections were carbon-coated by vacuum evaporation (SCD 004, Balzers, Lichtenstein). The SEM was set to an accelerating voltage of 20 kV and a working distance of 10 mm. The “Analyze particle” function of ImageJ (http://rsb.info.nih.gov/ij) was used to count the number of tubule cross sections in the electronic micrograph of the fractured tangential plane.

### Synchrotron radiation micro computed tomography

Synchrotron radiation micro-computed tomography (SR-μCT) measurements were performed at the BAM*line* at the synchrotron source BESSY II, Helmholtz-Zentrum Berlin für Materialien und Energien (HZB) [17, 18]. Samples were shaped into rods and were fixed in Kapton^®^ tubes with a diameter of 1, 2 or 4 mm, adapted to the size of the sample. The monochromatic radiation varied between 13.5 and 15 keV. For each CT scan, images at 1500 to 3000 angles per 180° were taken with individual exposure times ranging from 2 to 5 s. The volumes were reconstructed with a filtered-backprojection algorithm and visualized with the software VGStudio MAX 2.1 (VolumeGraphics GmbH, Heidelberg/Germany). The reconstructed volumes (from 5.10^−4^ to 40 mm^3^) had a voxel size between 0.4 and 1 μm. Virtual sections (2D images) of the samples and 3D visualizations of the tubular network were extracted for analysis. We performed two sets of preliminary SR-μCT experiments: one with analyzed volumes of about 40 mm^3^ with a resolution (voxel-size) of 1 μm and one with smaller volumes (5.10^−4^ mm^3^) with higher resolution of 0.4 μm. Combination of these two measurements provide an approximation of the tubule network.

### Chemical analysis

Quantitative micro-PIXE (Particle-Induced X-ray Emission) imaging of the surface of ivory sections was obtained at the external micro-beam line AGLAE (Accélérateur Grand Louvre d’Analyse Elémentaire, Paris, France), a 2 MV tandem accelerator (Pelletron 6SDH-2) [19, 20]. This technique allowed us to quantitatively map the major (Ca, P), minor (Mg) and trace elements (Na, Cl, K, S, Fe and Sr) of the surface of the ivory sections, which were fixed on a remote-controlled stage. The distance between the section and the exit nozzle was 3 mm. Data were extracted using the ListoEDF software [20]. For each pixel, low and high-energy X-ray spectra were extracted and quantitative analysis was performed with TRAUPIXE_EDF [20] and GUPIXWIN V2.1 software [21]. Finally the 2D colored maps were plotted with the R software (http://www.r-project.org./).

### 3D modeling

3D modeling of the tubular microstructure was performed in order to simulate the different 2D features resulting from the different angle of sectioning the tubules. The 3D software Rhinoceros (version 4 SR09) with the internal “Rhinoscript” scripting language was used (http://diva4rhino.com/). A detailed description of the different modeling is presented in S2 Fig and S1 Text and S2 Text and S3 Fig.

## Results

### The Schreger pattern varies in 3D

**Fig 2:**
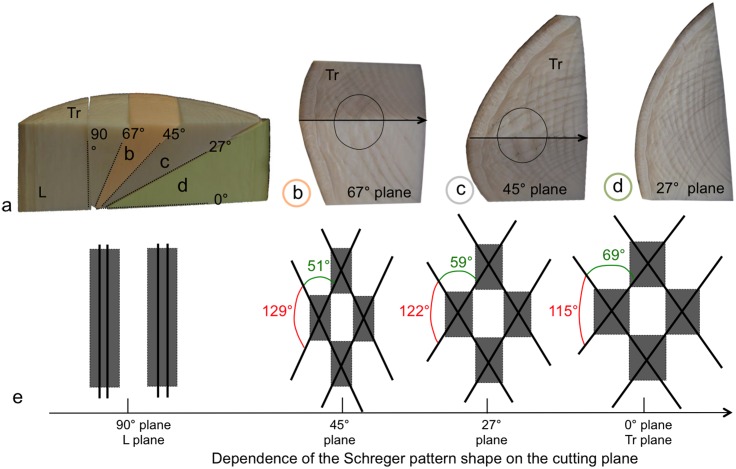
The Schreger pattern in 3D. (a) Cross-sections of the tusk cut at different angles (27°, 45° and 67°), (b-d) optical images of the surfaces of the 27°, 45° and 67° planes, (e) schematic variations of the shape of the Schreger pattern according to the cutting angle. The dark lines represent the Schreger lines and the gray forms represent the related Schreger rhomboid shapes.

Cross-sections of the elephant tusk at different cutting planes (67°, 45° and 27° relative to the transverse plane) show the variation of the Schreger pattern in 3D (Fig 2). As the sectioning angle decreased from 90° (longitudinal sections) to 0° (transverse sections) (Fig 2a), the parallel longitudinal dark and bright bands observed in longitudinal planes progressively crossed, forming a diamond-shaped honeycomb array characterized by the so-called Schreger angles, which vary in dimensions depending on the considered plane (Fig 2b, 2c and 2d). Each diamond shape presents two obtuse and two acute angles and so can be characterized by an obtuse/acute ratio: this ratio increased proportionally with the cutting angle, being 1.7, 2.1 and 2.5 respectively for the 0°, 27° and 45° planes (Fig 2e).

Moreover, although the Schreger angles vary between cutting planes, no interruption of the pattern was observed between sectioning planes in the same sample, with dark and bright bands continuous from one plane to another (as in the circles on Fig 2b and 2c, showing continuity of bands from the transverse section surface onto the 67° and 45° cutting planes, respectively). The Schreger pattern therefore changes continuously in 3D within the tusk.

### The Schreger pattern is related to the microtubule arrangement of the 2D planes

#### The transverse plane

As we found no relation between the Schreger pattern of the transverse plane and the chemical composition of ivory (Ca, P, Mg, Na, Cl, K, S, Fe, and Sr) from our micro-PIXE data (S4 Fig), we investigated the structural arrangement of the tubular network from three orthogonal cutting planes for correlations with the Schreger pattern.

**Fig 3:**
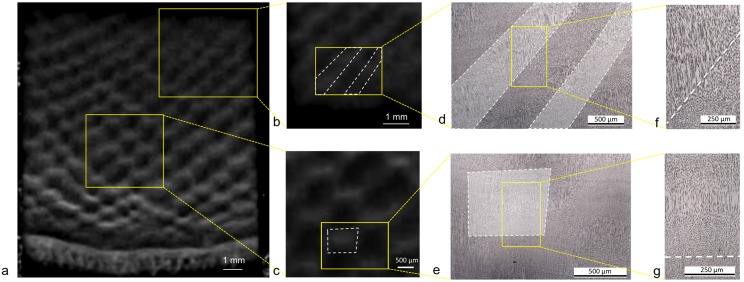
The Schreger pattern and the 2D tubular microstructure of the transverse plane. (a) Schreger pattern of polished transverse section of the tusk, (b) and (c) higher magnifications of (a), (d) and (e) related microstructure observed by reflected light microscopy, (f) and (g) higher magnifications of (d) and (e). The yellow rectangles show the location of the different magnifications and the white dotted lines indicate the bright area of the Schreger pattern.

Transverse sections of the tusk (Fig 3a) showed the Schreger pattern as described previously: staggered bright and dark rhomboids giving the impression of intersecting lines, radiating clockwise and anticlockwise from the tusk axis. Optical microscopy observations of polished sections allowed relation of the dark and bright areas of the Schreger pattern (Fig 3a) to the apparent microstructure (Fig 3d and 3e). Depending on sectioning angles of the tubules, tubular cross-sections appear as dark lines or dots (with elongated shape), surrounded by bright ivory matrix, with like features tending to cluster (i.e. lines with lines and dots with dots). In the majority of cases, dark Schreger areas corresponded to regions with many dots in higher magnification observations, whereas bright ones corresponded to regions with many lines (Fig 3d and 3f). In very few cases, bright areas contained both dot and line regions (shown above the white dashed line in the higher magnification image in Fig 3e and 3g).

In order to understand why regions of dots and regions of lines correspond to different colored areas at a macro-scale, respectively dark and bright, we investigated which characteristic of the tubular microstructure could be responsible for such difference.

**Fig 4:**
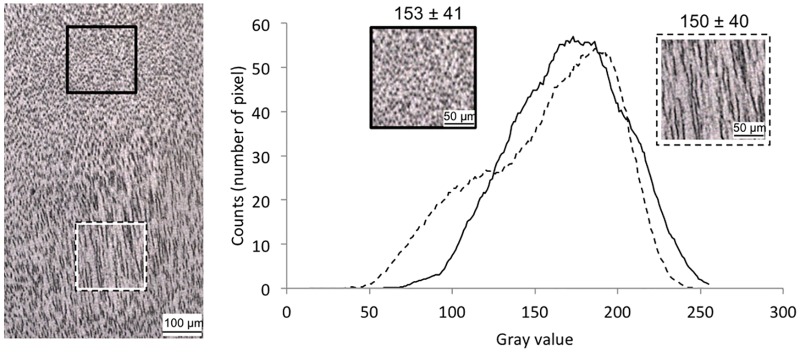
Comparison between the gray values of regions of dots versus regions of lines in the transverse plane. Histograms of the gray values of a region of dots and a region of lines (corresponding to dark and bright Schreger areas, respectively). Mean gray value and standard deviation of each selected area is indicated (0: black, 255: white).

First, we measured the mean and the distribution (histograms) of the gray scale values of the two regions (Fig 4). Both the mean values and the distributions of the gray scale values were very similar for the two regions (Fig 4). This means that the number of black and white pixels in the two regions was the same. Therefore, at a microscale, regions filled with dots or lines did not differ in apparent color.

Second, we investigated the spatial distribution of black and white pixels in the two regions. Contrary to gray values, the spatial distribution differs between dot and line regions because in one case black pixels form dots (isolated pixels) and in the other case, lines (joined pixels). This simply relates to the length of the black features. The length of the lines ranged from 10 to 50 μm whereas the mean diameter of the dots was 6 μm.

Third, we observed that dots were spaced more periodically than lines, which showed greater variation in their spacing. In order to quantify the difference in spacing of the two regions, we defined two parameters: the average line spacing (ls) in μm, which is the length of the optical image in μm divided by the number of lines, and the average dot spacing (ds) in μm, which is the square root of the area of the optical image in μm^2^ divided by the number of dots. For the transverse plane, ls was 17 μm, and ds 10 μm.

#### The longitudinal plane

**Fig 5:**
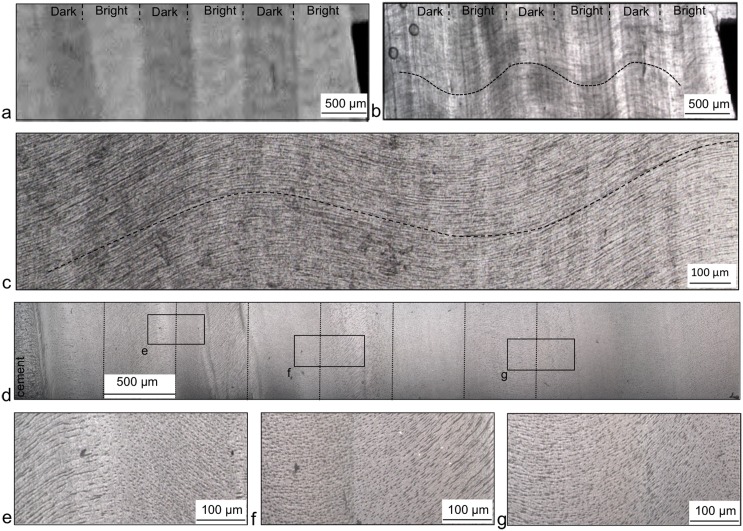
The Schreger pattern and the 2D tubular microstructure in the longitudinal plane. (a) photography of a thin section with the visible Schreger bands indicated, (b) observation of this same section by transmitted light with the sinusoidal trend of tubules drawn, (c) magnified sinusoid trend observed by transmitted light of a thin section, (d) thick section observed by reflected light where the dark and bright bands were hardly visible, (e-g) magnifications of the insets indicated in (d).

The features that can be observed in longitudinal planes depend on the thickness of the sections and the degree of polishing. Sections that were thin enough for light to pass through clearly showed the characteristic alternating Schreger bands of the longitudinal planes (Fig 5a). However, on polished thick sections, it was harder to distinguish the vertical bright and dark bands of the Schreger pattern. The thin sections, observed by transmitted light, showed interrupted lines with a maximum length of 200 μm, which had a sinusoidal trend (Fig 5b and 5c). The mean amplitude of the tubular trend was 230 μm and its mean wavelength 1160 μm.

Tubule microstructure was clearly visible in thick, polished sections observed under reflected light (Fig 5d, 5e, 5f and 5g). In most of the observations, alternating vertical bands (~500 μm wide) of 100 μm-long line regions and shorter line areas were identified. These vertical bands are the same width as the vertical Schreger banding seen at lower magnifications (e.g. Fig 1c).

### The microtubules’ arrangement in the 2D planes depends on the cutting angle

**Fig 6:**
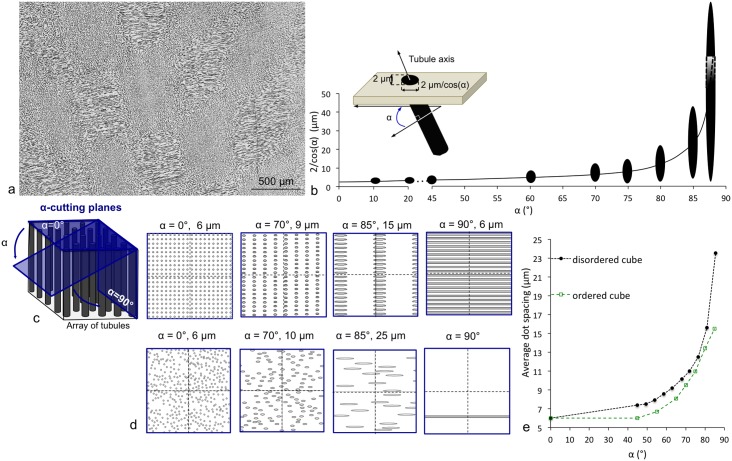
Relation between the orientation of tubules and the resulting shapes and average spacing of tubule cross-sections. (a) Experimental observation under reflected light microscopy of a polished ivory section of the transverse plane, (b) Variation of the length of the cross-section of tubules depending on the cutting angle α, (c and d) 2D projections of the sectioned tubules with 0°< α < 90° of (c) the ordered cube where tubules are periodic and (d) the disordered cube where tubules are non-periodic. Average dot and line spacing are indicated in μm (line spacing = 120 μm/number of lines and dot spacing = √ (14400 μm^2^/number of dots) and (e) plot of the average dot spacing versus α.

Cross-sections of microtubules arrange in regions of dots and lines in the transverse and longitudinal planes. The dot regions correspond to tubules cut oblique to their main axis whereas line regions to tubules cut along it. In particular, in the transverse plane, staggered rhomboid regions composed of packing of dots and lines were observed (Fig 6a). Moreover, a dot spacing ds_Tr_ = 10 μm was measured for the dot regions of the transverse plane. This value is different from the one measured for the tangential plane, where all tubules are cut perpendicularly to their main axis (ds_Ta_ = 6 μm) (see S2 Fig and S1 Text). This difference suggests that the sectioned tubules leading to the dot regions of the transverse plane were not cut at the same angle as the sectioned tubules leading to the feature of the tangential plane.

In order to understand the relationship between tubule cross-sectional shape and spacing with cutting plane, we investigated the shape and the average spacing of tubule cross-sections, with respect to a simulated cutting plane of sectioned tubules; we assume that at a small scale (few microns), tubules can be considered as straight tubes.

First, the relation between the length of the cross-section of one straight tubule and the cutting angle α at which this tubule was cut was established (Fig 6b). When α was 0°, the section was perpendicular to the main tubule axis and the tubule cross-section was a circle; as α increased, the cross-sections became longer ellipsoids until becoming an infinite line at α = 90°. The variation of cross-sectional shape was progressive until 70°, after which a very small change in the cutting angle induced a drastic change in the shape and size of the tubule cross-sections.

Second, two modeled cubes composed of periodic and non-periodic arrangement of many tubules were virtually cut by varying α in order to simulate the regions of dots and lines observed experimentally (Fig 6c) (see S2 Fig and S1 Text for details about the modeling). As for the results shown in Fig 6b, no significant changes of the tubular cross-section shapes and of the tubule average spacing were identified for α between 0 and 70°. Therefore, only results for α > 70°, exhibiting the most striking features, are presented for both cubes in Fig 6c and 6d. The average dot spacing for each simulated slice was then measured and is indicated next to the value of α. For α = 0°, all tubules were cut perpendicular to their main axis and the slice gave a periodic arrangement of dots for the ordered cube and a irregular one for the disordered cube corresponding to the experimental data of the tangential plane with an average spacing of 6 μm (see S2 Fig and S1 Text). For α = 70°, the slice of the ordered cube gave ds = 9 μm, different from the one of the experimental data (10 μm) whereas the slice of the disordered cube had a ds = 10 μm, corresponding to the experimental data (Fig 6d). In the experimental data, the specific arrangement of tubules in 3D together with an angle of cut of 70° reproduce the 10 μm tubular spacing of the experimental observation of the transverse plane. Therefore, tubules that appear as dots in the transverse plane were likely inclined at 70° to the sectioning plane.

Finally, the relation between α and the average dot spacing for the ordered and disordered cube (Fig 6e) shows that both the shape of the cross-section and the average dot spacing vary similarly according to α, with an inflexion point of the curve between 70° and 80°, after which changes occur more rapidly.

Our experimental data and the two previous simulations show that the different regions of the 2D tubular microstructure (dots and lines) observed at the surface of ivory section, can be characterized by three parameters: 1) an average spacing of the tubular cross-sections, 2) a mean length of the tubular cross-section and 3) a specific orientation of the tubular main axis to the cutting plane. Because these three parameters are related to each other, if one is experimentally known, the others can be calculated. For example, thanks to the experimentally measured ds = 10 μm for the dot regions of the transverse plane, we calculated an inclination of 70° of the tubules to the plane in these regions and a length of the tubular cross-sections of 6 μm in the transverse plane.

### The helical shape of tubules

#### The 2D observations

In the transverse plane, close to the cement, a sinusoidal trend of the tubules can be tracked through the regions of lines and dots (S5 Fig). The mean amplitude of the tubular trend is 90 μm and its mean wavelength is 960 μm. In the longitudinal plane we also observed a sinusoidal trend (Fig 5). Therefore, two sinusoid trends in two orthogonal planes (L and Ta) could suggest the presence of a helical tubule in 3D. (For details of the equation of the helix see S2 Text).

**Fig 7:**
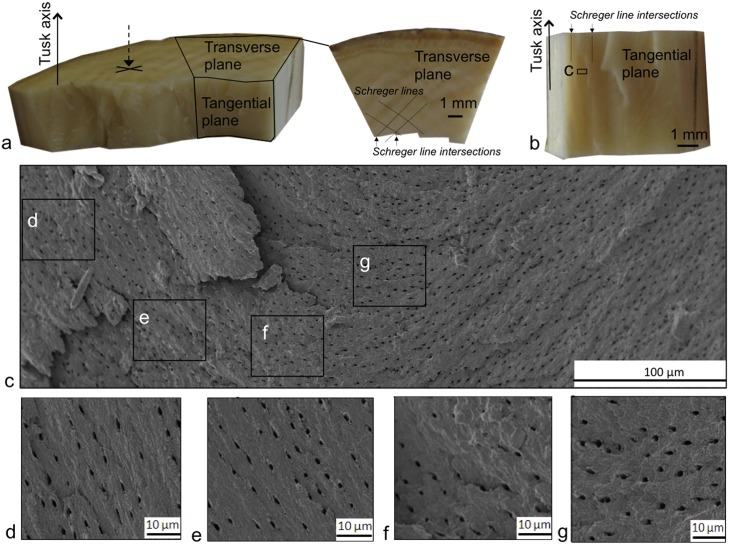
The 2D tubular microstructure of the tangential plane. (a) Fractured section of the elephant tusk (1.6 cm away from the cement) with the impact of the fracture represented by the cross and the arrow, the transverse and tangential planes indicated and the Schreger lines and the Schreger line intersections of the transverse plane also displayed, (b) magnification of the tangential plane of (a), (c) area indicated in b between two Schreger line intersections and observed under SEM, and (d-g) magnification of the zones indicated in c.

Moreover, transverse sections of the elephant tusk were fractured along the tangential plane (Fig 7a and 7b). The transverse plane presents the typical Schreger lines (Fig 7a). The tangential fractured surface observed under SEM shows that the tubular cross-section shapes and 2D arrangement are the same every 1mm. Areas of 500 μm between two Schreger line intersections show in detail this arrangement and the shape of tubule cross-sections (Fig 7c), which are ellipsoidal in shape, 2 μm long, and 1.2 μm wide on average. The ellipsoids progressively changed their orientations from an inclination of 20° to the vertical of the sample on the left-hand side (Fig 7d) to an inclination of 90° (Fig 7g). This change of orientation had a periodicity of 1 mm, which is represented in Figure A in S6 Fig. The periodic change of orientation of the ellipsoid tubular cross-sections strongly suggests a continuous phase shift of π every 1 mm in the tangential direction of helical tubules (see Figure B in S6 Fig for more details).

#### The 3D data

Our 2D data suggest that tubules are ~2 μm in diameter, and have a helical form with a pitch of ~1 mm and are ~6 μm spaced. In order to visualize the expected helices in 3D and discern separated tubules, analyses of large volumes with high resolution were needed.

According to the lower resolution SR-μCT experiments a helical trend of the average orientation of the main direction of several tubules was calculated with a pitch of about 1 to 1.5 mm (S7 Fig). However, the resolution here was not adequate to allow a 3D reconstruction of individual tubules, which was possible with the higher resolution SR-μCT experiments. In this latter experiment, the tubular network was reconstructed in 3D and bridges (or connections) that often connect tubules were visualized (S8 Fig). Tubules appeared curved, but the analyzed volume was here too small to be able to identify a completed helical trend.

## Discussion

Because of its striking appearance and apparent complexity, the source of the Schreger pattern has fueled debates in the literature for decades [2–6, 16]. Biological colors can arise from a variety of mechanisms, either from pigments, as for mollusk shells, or from the interaction of light with surface structure, as with nacre, some bird feathers, butterfly wings, and chameleon skin [22, 23]. The origin of the Schreger pattern has been assumed to stem from the tusk dentine’s tubular microstructure in all previous studies [2–6] except one [16], which believed the arrangement of mineralized collagen fibers to be the source. However, no convincing relation between the macroscopic Schreger pattern and ivory microstructure, either tubular or fibrous, was ever previously clearly established.

### Relation between the macroscopic Schreger pattern and the ivory 2D microstructure: Where does the color come from?

Here, we addressed the nature of the Schreger patterning, by first showing that the chemical composition is unlikely to be involved in its formation, as major constituent elements of the ivory are distributed uniformly over large areas. Second, we established that, on average, in transverse sections of elephant tusk, the dark areas of the pattern correspond to tubules oblique to the transverse plane at 70° (appearing as dots) whereas bright areas relate to tubules aligned with it (appearing as lines). The size and the staggered arrangement of dot and line regions correspond well in general with the colored checkerboard pattern, supporting previous assertions [2–6] that tubule microstructure dictates Schreger patterning. However, it is worth noticing that the pattern has been simplified and abstracted for years, described by the majority of authors as a perfect checkerboard pattern [2–5]. We observed, however, that some heterogeneities do exist, that the pattern is not as periodic and perfect as claimed to be at both the macroscopic and the microscopic scales and the exact correspondence between the 2D microstructure and the Schreger pattern is sometimes missing. For example, the bright zones of the Schreger pattern of the transverse plane sometimes relate to areas with dots in the middle of the rhomboids and lines at their periphery (e.g. Fig 3e and 3g).

In longitudinal sections, periodic elongated dot and line regions also seem to correspond to alternating dark and bright bands of the Schreger pattern, respectively. By analogy to the transverse plane, line regions correspond to bright bands and dot ones to dark bands. Additionally, we showed that the Schreger pattern is not a phenomenon isolated to 2D sectioning planes, but rather a continuous 3D pattern throughout the elephant tusk, with the pattern uninterrupted from one plane to another, although the Schreger angles change with cutting plane. Schreger angles vary within the transverse section from cement to pulp and depend on the location of the transverse section along the tusk axis [10–13]. Here, we additionally show that Schreger angles vary in 3D. Most of the carved ivory art objects present strong reliefs. Therefore, Schreger angles observed at the surface of such objects do not only depend on the Elephantoidea taxa but also on where in a tusk objects were crafted, which is usually unknown. Thus, Schreger angles must be used very carefully for identifying different types of Elephantoidea ivory on carved objects and additional methods should be use to efficiently fight against illegal trade of ivory.

The physical reasons as to why regions of dots and lines produce dark and bright areas, respectively, require further investigation. However, we first showed that the average area fraction of tubules intersected is the same in regions of dots and lines. In other words, thresholded images of line or dot regions into black and white images, with black corresponding to regions inside the tubule, showed constant average density of black pixels. This shows that differences in tubule density cannot be responsible for the emergence of the Schreger pattern as proposed by some authors [3, 4]. Second, we determined three 2D microtubular characteristics, which differ between the two regions of dots and lines: the average spacing, the length of the tubular cross-section and the orientation of the tubular main axis to the cutting plane. However, the difference of spacing between areas of dots (10 μm) and lines (17 μm) and the difference of length of the tubule cross-sections (10–50 μm for lines, and 6 μm for dots) found in the transverse plane also do not seem to be the origin of the Schreger pattern. Indeed, these differences are too small to be seen by the human eye and yet too large compared to the wavelength of light to be at the origin of any sort of structural color.

These two points imply that other characteristics might be responsible for the differences in color observed. One could be correlated to tubule orientation, as for example the interaction of impinging light with ivory material and its specific microstructure (empty tubules specifically oriented to the cutting plane) (see S9 Fig and S3 Text for more details). A second one could be the amount and type of bright-dark interfaces between the empty tubules (dark) and the ivory matrix (bright) in the two types of regions. Indeed, our perception of grey scales is strongly related to the presence of interfaces between dark and bright according to the well-known Cornsweet illusion [24]. Although an analysis of this effect goes well beyond the scope of the paper, it is not excluded that the brighter appearance of the line regions relative to the dot regions may be due to this optical effect. Indeed, these interfaces are very different in amount and structure between the two regions. In regions of lines, they are nearly straight and clearly visible whereas in the dot regions they are less pronounced, which could lead to line regions appearing brighter than dot ones.

### 3D models of the ivory microstructure

Our 2D experimental observations can be summarized by one key feature for each of the three planes of a small volume of the mid-dentin of the tusk: 1) the structural staggered pattern with separated rhomboids of dots and lines of 500 x 500 μm^2^ for the transverse plane, 2) alternating vertical bands (~500 μm wide) of 100 μm-long line regions and elongated dot areas having a sinusoidal trend (wavelength of 1 mm) for the longitudinal plane and 3) the periodic change of orientation of the ellipsoidal cross-sections every 1 mm for the tangential plane. Because the Miles and White and the Virág models failed to describe our experimental data obtained on modern elephant ivory samples, we proposed a third model with helical tubules, having a combination of a continuous shift of π after 1 mm with a stepwise shift of π/2 after 500 μm.

**Fig 8:**
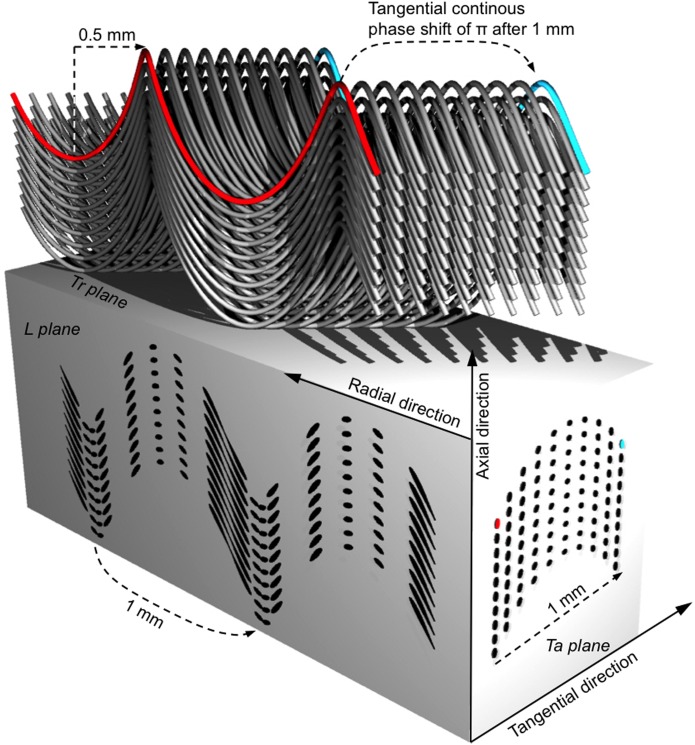
Representation of the 3D distorted helical model. A set of helical tubules is represented. For the sake of the visualization of the model the diameter of the tubules and the spacing between them have been increased times 5 and 10 respectively. However, the dimension of the helices and the continuous phase shift of π after 1 mm are properly scaled. From the red tubule to the turquoise one the continuous phase shift of π after 1 mm is observed.

Whereas most previous models suggested sinusoidal tubules [2, 6], our results succeed in extending the understanding of tubule morphology into three dimensions. Both our 2D observations on tubular cross-sectional shape from the three orthogonal planes and our 3D SR-μCT data suggest that tubules exhibit a helical shape. The dimensions of the helix have been determined according to the amplitudes and periods of the sinusoidal trends observed in the longitudinal and transverse planes. The longitudinal component of the helix is 2.5 times larger than the tangential one. The pitch of the helix (radial component) is about 1 mm and its amplitude (axial component) 230 μm long. Therefore, the helical shape of tubules is not regular but rather distorted. A 3D model of the distorted helical model is shown Fig 8. The sections of the 3D model along the longitudinal and tangential plane are also represented, they show the characteristic experimental features observed for these planes and further discussed below. Other views of the 3D model are shown Figure C in S6 Fig.

The proposed distorted helical shape of tubules in the case of elephant ivory, which reflect odontoblasts migration [25] during ivory formation, might be viewed as a non-economical way for the cells to proceed. However, tubules in mammalian dentine present as the simplest shape, s-shapes [26]. This curved movement has been proposed to be due to the crowding of the odontoblasts as they move from the periphery toward the pulp inducing intercellular pressure [4]. Recently, genetic regulations in Elephantoidea have also been suggested to be responsible for sinusoidal tubules [6]. In dental enamel, wavy structures are also very common [27] and recent simulation work has suggested that the curved migration paths of ameloblasts could be the result of strain stimulus [28]. Finally, not only curved but also helical movements are common in nature, from molecular to macroscopic levels. Chirality exists at different levels of organization in biological materials such as plant tendrils and snail shell [29, 30]. The micro-chirality of the tubule morphology might be the biological response to particular mechanical stress and biological functions. The precise chirality, left or right handed of the cell path still remain to be determined.

The idea that ivory tubules are phase shifted is not new: Although no experimental data were presented, Miles and White [2] proposed, in a diagrammatic cross-section of modern elephant tusk, that sinusoidal tubules radiate with a stepwise phase shift of π in the tangential direction every 500 μm (S1 Fig). Virág [6] expanded on the Miles and White model, proposing an additional continuous phase shift of π between tubules every 1 mm also in the tangential direction, based on optical and electron microscopy observations in the three planes (transverse, longitudinal and tangential) from thin and fractured sections of mammoth and extinct elephant ivory. Our results in tangential sections support Virág’s model concerning the continuous phase shift of π every 1 mm in the tangential direction (S6 Fig). How and why cells create such a shift is a very interesting biological question which would need further studies. For example, the regular wrinkled cement-dentin junction observed on transverse section could be related to the phase shift (Fig 1b).

Finally, the staggered 2D microstructure observed on the transverse plane is likely due to the presence of a stepwise phase shift of π/2 in the tangential direction every 500 μm (contrary to the one of π in the Miles and White model).

**Fig 9:**
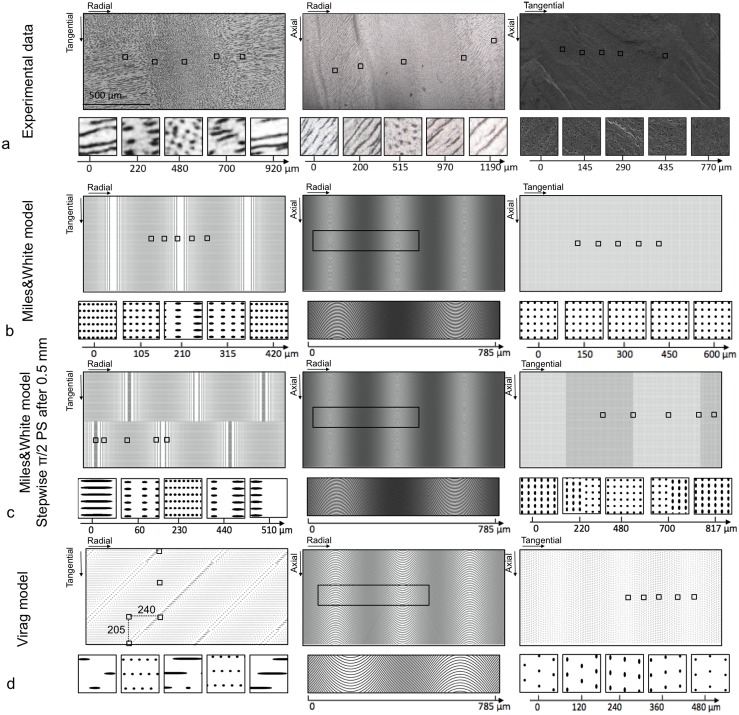
2D sections of the transverse, the longitudinal and the tangential planes of (a) the experimental data, (b) the Miles and White model and (c) the Miles and White model with a phase shift (PS) of π/2 after 500 μm instead of π and (d) the Virág model. The parameters used for Miles and White model were sinusoids with 250 sin(2π / 1000t) as equation with a diameter of 2 μm, 6 μm spaced and having one stepwise tubule PS of π after 500 μm; for Virág model: sinusoids with 300 sin(2π / 1000 t) as equation with a diameter of 2 μm, spaced by 13 μm and having one continuous PS of π after 1 mm and a stepwise one of π after 500 μm. Under every 2D section five magnified areas (34 x 34 μm^2^) are shown.

**Fig 10:**
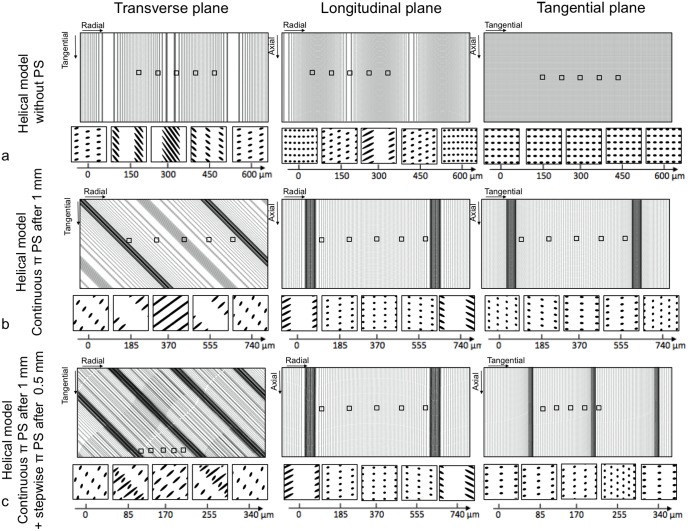
2D sections of the transverse, the longitudinal and the tangential planes of the helical model. (a) without phase shift (PS) of the tubules, (b) with continuous PS of π after 1 mm and (c) with both a continuous PS of π after 1 mm and a stepwise PS of π after 500 μm.

Although Miles and White [2] and Virág [6] attempted to reconcile existing 2D microstructural observations with Schreger patterning by proposing 3D models of tubule arrangements, they were not able to establish clear links between features of 3D tubule morphology and the pattern that would result from sectioning them. In Figs 9 and 10, we compare our experimental data (Fig 9a) with virtual slices from a series of 3D models (Figs 9b, 9c and 10), including those of Miles and White and of Virág, as well as several new models with helical tubules, which vary in terms of the nature of the phase shift of their tubules (for a detailed description of the models, see S2 Text and S3 Fig). To assist in the comparison, the five most striking areas (32 x 32 μm^2^) of each section were magnified in order to provide a detailed view of the microstructure, namely the shape and size of the tubule cross-sections and their arrangements (for a detailed description see S4 Text).

Table 1 summarizes relevant parameters from all 3D model cross-sections, as a means of contrasting each model with our experimental data. Slices from our suggested 3D helical models produced morphological parameters that were most similar to the experimental ones (bold parameters in Table 1), with the helical model with continuous phase shift of π best reproducing the experimental observations. Although we did not find the perfect match between 2D experimental observations and the helical model, we now better understand the role of the shape of the tubule and their phase shift on the 2D tubular microstructure in the three planes:

1. The staggering observed in the transverse plane has been only reproduced by a stepwise phase shift of π/2 after a distance of ½ of the tubular wavelength.
2. Although the periodicity of the line (and dot) regions of 1 mm has not been perfectly reproduced with a tubular wavelength of 1 mm, the combination of helical tubules and a continuous phase shift of π after a distance of the tubular wavelength (1 mm) allowed the model to approximate this periodicity.
3. The same combination (helical tubules and phase shift π after 1 mm) reproduces the prominent feature we observed in the tangential plane, where the orientation of the ellipsoidal tubule cross-sections changed every 1 mm.

**Table 1:** Summary table of the experimental parameters compared to the ones obtained with the different 3D models.

**Parameters**			**3D Models**					
			***Miles and White***	***Modified Miles and White***	***Virag***	***Helical***		
	**Parameters of the 3D models**	Tubule shape	Sinusoidal	Sinusoidal	Sinusoidal	Helical	Helical	Helical
		Stepwise phase shift	π after 0.5mm	π/2 after 0.5mm	π after 0.5mm	-	-	π after 0.5mm
		Continuous phase shift	-	-	π after 1mm	-	π after 1mm	π after 1mm
**Parameters of the 2D planes**		***2D Experimental data***	**2D simulated slices of the 3D models**					
			***Miles and White***	***Modified Miles and White***	***Virag***	***Helical***		
**Transverse plane**	Staggering	Yes	No	Yes	No	No	No	No
	Periodicity of line (and dot) regions	1mm	0.5mm	0.5mm	0.5mm	0.5mm	**0.75mm**	-
	Width of line regions	0.5mm	0.12mm	0.12mm	0.1mm	0.25mm	**0.38mm**	-
	Length of lines	10–50μm	8μm	30μm	25μm	30μm	**45μm**	-
	Overlaps	No	No	No	No	No	Yes	Yes
**Longitudinal plane**	Sinusoidal trend	Yes	Yes	Yes	Yes	Yes	Yes	Yes
	Periodicity of lines (and dot) regions	1mm	-	-	-	0.3mm	**0.75mm**	**0.75mm**
	Width of line regions	0.5mm	-	-	-	0.18mm	**0.38mm**	**0.38mm**
	Length of lines	0.1mm	-	-	-	10μm	**10μm**	**10μm**
	Overlaps	No	No	No	No	No	Yes	Yes
**Tangential plane**	Transverse cross-sections	Ellipsoids	Circular	Circular and ellipsoids	Circular and ellipsoids	**Ellipsoids**	**Ellipsoids**	**Ellipsoids**
	Change in shape	No	No	Yes, stepwise	Yes, progressive	**No**	**No**	**No**
	Change in orientation	Yes progressive	No	No	No	**No**	**Yes Progressive**	**Yes Progressive**
	Periodicity	1mm	-	1mm	0.5mm	-	1mm	0.35mm
	Overlaps	No	No	No	No	No	Yes	Yes

In bold, the most similar parameters obtained with the model compared to the experimental data.

The simulated arrays of helical tubules showed overlaps that are only possible if tubules are more interconnected than originally thought. This overlap in the tubules arises when the average spacing of tubules is sufficiently small, so that a phase shift causes the spirals of the helices to intersect, highlighting the geometric limitations in packing spirals in 3D. To resolve this, detailed 3D imaging would be required in order to see the packing of tubules in a narrow but long volume of ivory.

Here, our 3D model, based on experimental data arising from a variety of high resolution characterization techniques, focuses on Schreger pattern features in a small volume of the mid-dentin from the middle of the tusk. However, one needs to remember that ivory originates from very large biological structures, tusks, where heterogeneities in 3D over longer length scales are surely present. Indeed Palombo [10] and Virág [6] mentioned the variation of the wavelength of sinusoidal tubules from cement to pulp by showing that the wavelength increases toward the pulp cavity. This is supported by our observations of the variation in Schreger rhomboid shape and dimensions from cement to pulp (S1 Fig). The variation in the longitudinal direction appears to be much less investigated, due to the technical challenges in analyzing microscopic scale features over macroscopic length scales. Given recent advances in the accessibility of high-resolution materials characterization techniques, it is hoped that a more detailed understanding of how these complex tissues vary and grow in 3D will soon be possible.

### Conclusions

Most of the previous studies hypothesized that the Schreger pattern originates from the organization of the 3D tubular network, although no evidence has ever been proposed to our knowledge. By computational approaches, we demonstrate that previous models do not completely reproduce the experimental micro-tubular arrangement in the three orthogonal planes (transverse, longitudinal and tangential). Employing systematic observations of the three planes of the same ivory section from macro down to micro scale, and by using complementary analytical methods (SEM, LM in reflection and transmission and SR-μCT), we identified previous inconsistencies and filled gaps in our understanding of first, the 3D tubular arrangement of elephant ivory and second, its possible role into the origin of the Schreger pattern.

First, we found that helical tubules having phase shift of π after 1 mm in the tangential direction (phase shift n°1) combined with a π/2 stepwise shift after 500 μm also in the tangential direction (phase shift n°2) better represent the 2D tubular arrangement observed experimentally in the three planes. Phase shift n°1 is responsible for the changes in orientation of the ellipsoid tubular cross-sections in the tangential plane whereas phase shift n°2 is responsible for the emergence of the staggered microstructure of the transverse plane. Phase shift n°2 is different from the one proposed by Miles and White [2] to be at the origin of the staggered Schreger pattern. Both, the helical shape of tubules (with a 1 mm wavelength) and the phase shift n°1 allow better approximation of the experimental periodicity of the staggered tubular microstructure in the transverse plane. This lack of correspondence between the 1 mm tubular wavelength and the microstructural periodicity observed in the transverse plane was one of the main inconsistencies of the previous 3D models [2, 6].

Second, for the first time we clearly determined the direct relation between the Schreger pattern and the 2D microtubule arrangement. Dark areas of the Schreger pattern mainly correspond to regions where tubules are cut obliquely to their main axis, whereas bright areas mainly correspond to tubules cut along their main axis. This established relation between the pattern and the structure as well as the determination of different microtubular characteristics are very important starting points for future investigations of the origin of the Schreger pattern.

In general, our findings contribute not only to our understanding of tubule morphology and its association with the Schreger pattern, but also to our knowledge on tissue growth mechanisms of elephant ivory, as tubules are remaining evidence of the odontoblast path during ivory formation. Moreover, thanks to our better appreciation of how the Schreger pattern is distributed and varies in 3D, we advise that the Schreger angles alone are insufficient for identifying different kind of Elephantoidea ivory used in carved objects, as suggested by Espinoza and Mann [3, 14]. Future studies, incorporating structural and chemical variations over macroscopic length scales, will be necessary for developing such diagnostic tools.

## Supporting Information

S1 Doc Document for the acquisition of the elephant tusk. (PDF)

S1 Fig Schreger pattern of the transverse plane. (PDF)

S2 Fig Average dot spacing and coordinates of tubule cross-sections of a tangential fractured section of elephant ivory and description of the modeling of the two cubes of straight tubules. (PDF)

S3 Fig Virtual cut through an array of helical (or sinusoidal) tubules obtained through a series of successive cuts through individual tubules. (PDF)

S4 Fig Quantitative 2D elemental chemical maps of the transverse section of elephant ivory. (PDF)

S5 Fig Tubular sinusoidal trend of the transverse plane. (PDF)

S6 Fig Tubular cross-section arrangement and shape in the tangential plane. (PDF)

S7 Fig SR-μCT data of the 40 mm^3^ volume with 1 μm resolution (voxel-size). (PDF)

S8 Fig SR-μCT data of the 5.10^−4^ mm^3^ volume with 0.4 μm resolution (voxel-size). (PDF)

S9 Fig Influence of light on the origin of the Schreger pattern. (PDF)

S1 Text Details on the modeling of the two cubes. (PDF)

S2 Text Modeling. (PDF)

S3 Text Influence of light on the origin of the Schreger pattern. (PDF)

S4 Text Detailed description of the 2D slices obtained by virtually cutting the 3D models. (PDF)
