# Supplementary material for: Relation between the Macroscopic Pattern of Elephant Ivory and Its Three-Dimensional Micro-Tubular Network
Source: PLoS One. 2017 Jan 26;12(1):e0166671. doi: 10.1371/journal.pone.0166671 (PMC5268646; doi:10.1371/journal.pone.0166671)
Supplement: S1 Doc — (PDF) [file pone.0166671.s001.pdf]

**MINISTERE DU BUDGET**

**direction générale**
**des douanes**
**et droits indirects**

" Copie pour information "

Sous direction
des affaires juridiques
et contentieuses

PROCES VERBAL

DE CESSION

Bureau D/2

24 JUIN 1993

Nº 0 1 6 5 2

En vertu des dispositions de l'article 390 § 1 du code des douanes et de l'article 6 § 3-c de l'arrêté du 26 septembre 1949, les marchandises suivantes, abandonnées après transaction au profit de l'administration des douanes sont remises à titre gracieux à Monsieur le Directeur des Musées de France:

- deux défenses d'éléphant entières, non travaillées, de 140 cm de longueur et d'un poids respectif de 20 Kgs 200 et 20 Kgs 300,

- deux défenses d'éléphant de 73 et 78 cm, d'un poids total de 6 Kgs.

Paris, le
Le directeur général des
douanes et droits indirects,

Je soussigné, Monsieur Jacques SALLOIS, Directeur des Musées de France, reconnais avoir reçu les objets visés ci-dessus. qui seront inscrits à l'inventaire du laboratoire des musées de France

Paris, le
Le directeur des Musées de France,

23 bis, rue de l'Université - 75700 PARIS - Télex FIDOUAN A et B nº 220 200 - Tél : (1) 40.04.04.04
