# Supplementary material for: Relation between the Macroscopic Pattern of Elephant Ivory and Its Three-Dimensional Micro-Tubular Network
Source: PLoS One. 2017 Jan 26;12(1):e0166671. doi: 10.1371/journal.pone.0166671 (PMC5268646; doi:10.1371/journal.pone.0166671)
Supplement: S1 Fig — (PDF) [file pone.0166671.s002.pdf]

S1 Figs.

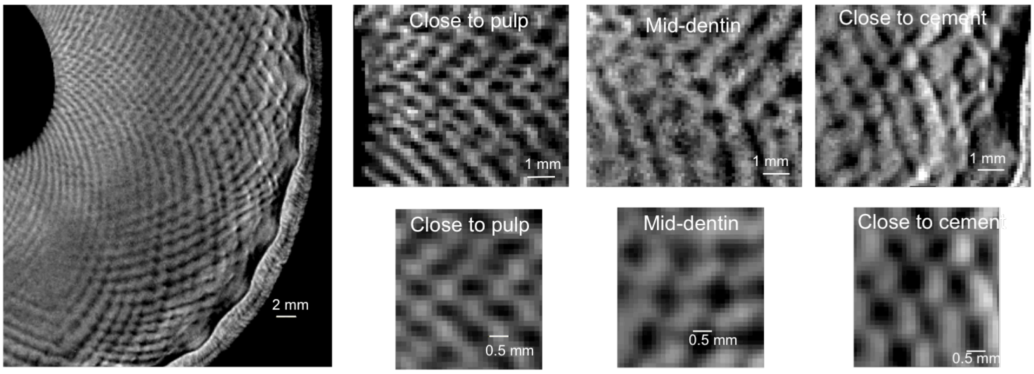

Figure A: Schreger pattern of the transverse plane difference in the rhomboids shape from cement to pulp. Close to the pulp the rhomboids are rectangles (0.2 x 0.5 mm^2) with their short dimension aligned along the pulp cavity, in the mid-dentin they have squared shape (0.5 x 0.5 mm^2) and close to the cement they are rectangles with their longest dimension along the

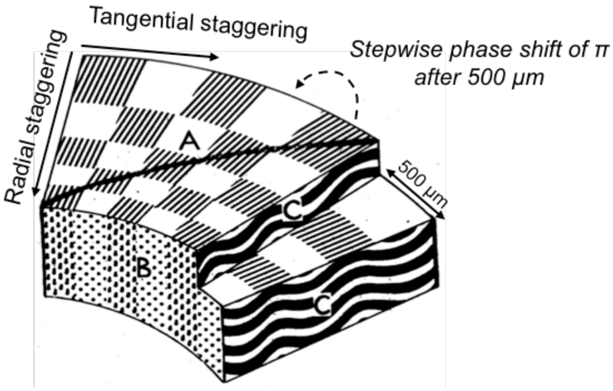

Figure B: Miles and White (1960) 3D model of the tubular microstructure with the radial and tangential staggering indicated and the phase shift of π after 500 µm

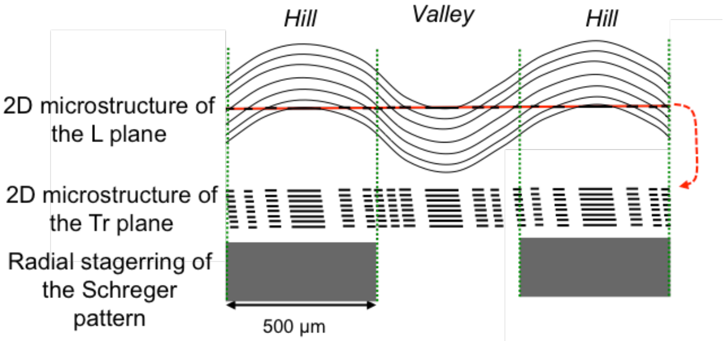

Figure C: Relation between the macroscopic Schreger pattern of the transverse plane and the tubular microstructure. According to Miles and White model, hills of the tubules correspond to the dark rhomboids of the Schreger pattern of the transverse plane, and valleys relate to the bright ones. Once sinusoidal tubules are cut perpendicular to their main axis, ie. in the transverse plane, the 2D microstructure results in an alternating regions of short and long lines. The 2D microstructure of the transverse plane is the same in dark and bright rhomboids according to Miles and White model.
