# Supplementary material for: Relation between the Macroscopic Pattern of Elephant Ivory and Its Three-Dimensional Micro-Tubular Network
Source: PLoS One. 2017 Jan 26;12(1):e0166671. doi: 10.1371/journal.pone.0166671 (PMC5268646; doi:10.1371/journal.pone.0166671)
Supplement: S2 Fig — (PDF) [file pone.0166671.s003.pdf]

S2 Figs. Average dot spacing and coordinates of tubule cross-sections of a tangential fractured section of elephant ivory and description of the modeling of the two cubes of straight tubules.

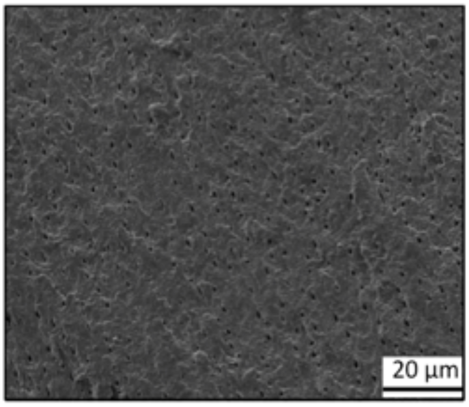

Figure A: SEM micrograph

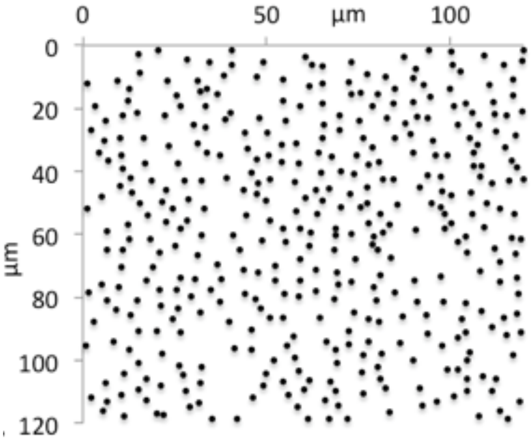

Figure B: Measured coordinates of every tubule cross-sections used to simulate the disordered cube.
