# Supplementary material for: Relation between the Macroscopic Pattern of Elephant Ivory and Its Three-Dimensional Micro-Tubular Network
Source: PLoS One. 2017 Jan 26;12(1):e0166671. doi: 10.1371/journal.pone.0166671 (PMC5268646; doi:10.1371/journal.pone.0166671)

S3 Fig. Virtual cut through an array of helical (or sinusoidal) tubules obtained through a series of successive cuts through individual tubules. Simulation of the intersection of many tubules with a cutting plane (i and vi) by calculation of the cross-sections in an iterative manner. The intersection of each individual tubule was calculated (ii-iii), and added to the intersection of the next tubule which is translated a known distance from the previous one (iv-v).

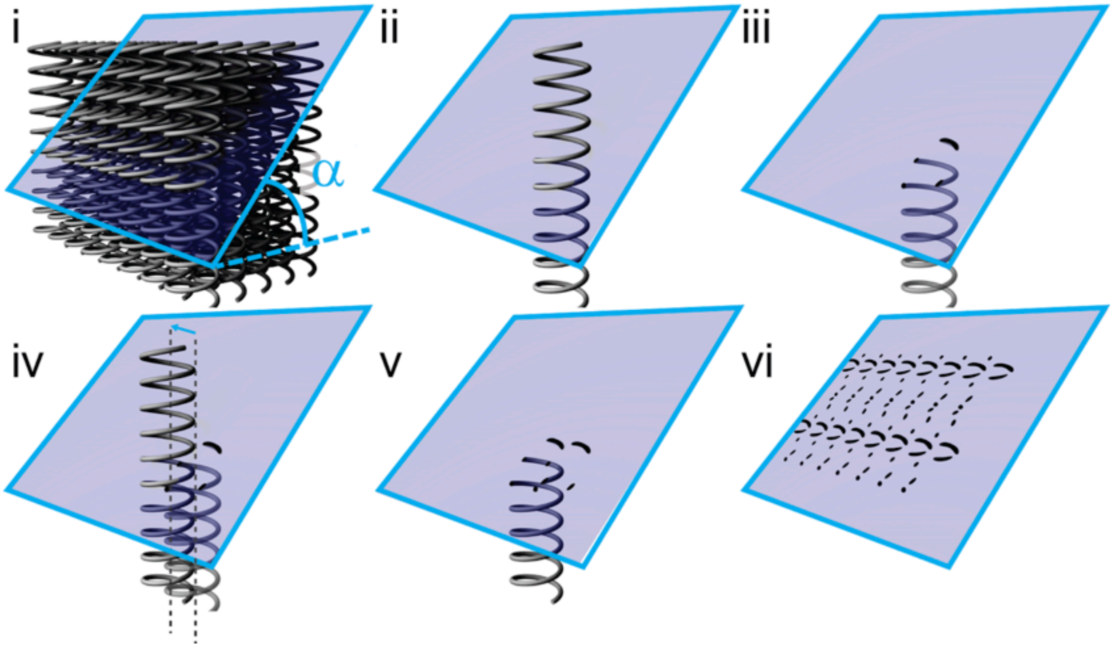

Supplement: S3 Fig — (PDF) [file pone.0166671.s004.pdf]
