# Supplementary material for: Relation between the Macroscopic Pattern of Elephant Ivory and Its Three-Dimensional Micro-Tubular Network
Source: PLoS One. 2017 Jan 26;12(1):e0166671. doi: 10.1371/journal.pone.0166671 (PMC5268646; doi:10.1371/journal.pone.0166671)

S4 Fig. Quantitative 2D elemental chemical maps of the transverse section of elephant ivory. 2D maps of 3 x 3 mm^2 of major elements of ivory (Ca and P), minor element (Mg) and traces (Na, S, Cl, K, Fe and Sr) obtained by Proton induced x-ray emission. Concentrations are expressed in wt.%.

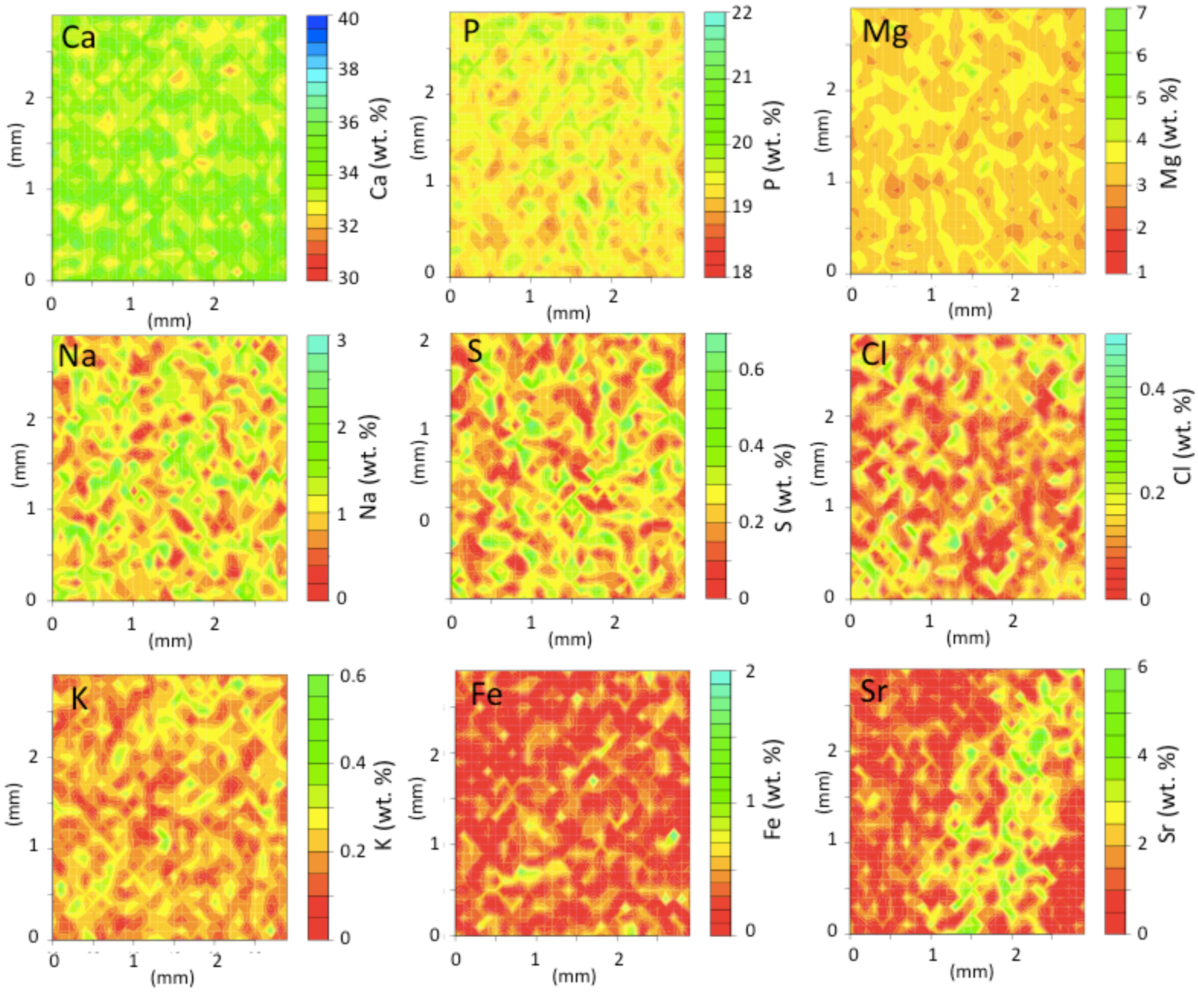

Supplement: S4 Fig — (PDF) [file pone.0166671.s005.pdf]
