# Supplementary material for: Relation between the Macroscopic Pattern of Elephant Ivory and Its Three-Dimensional Micro-Tubular Network
Source: PLoS One. 2017 Jan 26;12(1):e0166671. doi: 10.1371/journal.pone.0166671 (PMC5268646; doi:10.1371/journal.pone.0166671)

S5 Fig. Tubular sinusoidal trend of the transverse plane. Optical image of a thick polished section of the transverse plane located close to the cement. The mean amplitude of the tubular trend is 90 µm and its mean wavelength is 960 µm.

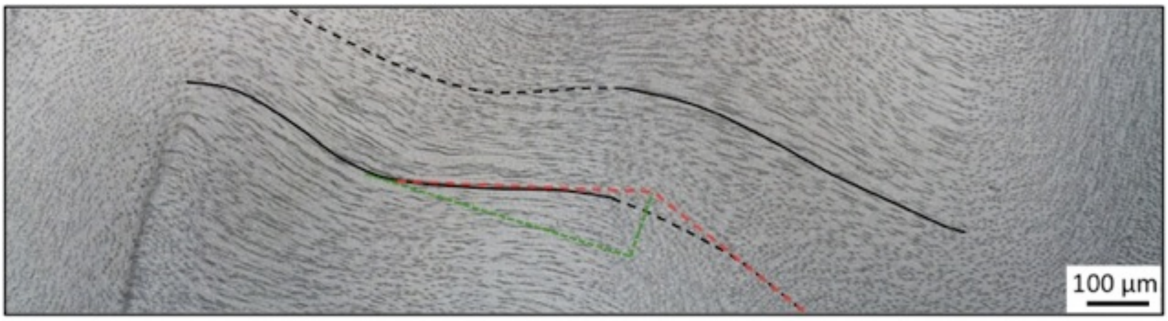

Supplement: S5 Fig — (PDF) [file pone.0166671.s006.pdf]
