# Supplementary material for: Relation between the Macroscopic Pattern of Elephant Ivory and Its Three-Dimensional Micro-Tubular Network
Source: PLoS One. 2017 Jan 26;12(1):e0166671. doi: 10.1371/journal.pone.0166671 (PMC5268646; doi:10.1371/journal.pone.0166671)
Supplement: S6 Fig — (PDF) [file pone.0166671.s007.pdf]

S6 Figs. Tubular cross-section arrangement and shape in the tangential plane.

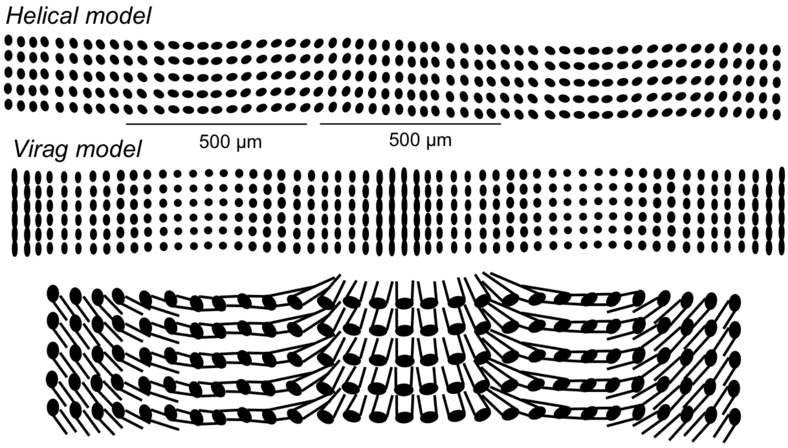

Figure A: Representation of our experimental observations and the one of Virag's model.

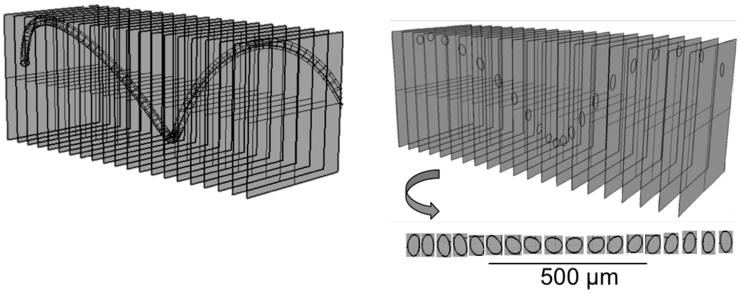

Figure B: Visualization of the shifted helical tubules behind the tubular cross-sections and simulated 2D planes of cut of the helix with the resulting cross-sections under every cutting plane. The obtained cross-sections have the same shape and orientation of the experimental data, suggesting that shifted helical tubules might likely be at the origin of the specific arrangement and shape of the tubular cross-sections observed in the tangential plane.

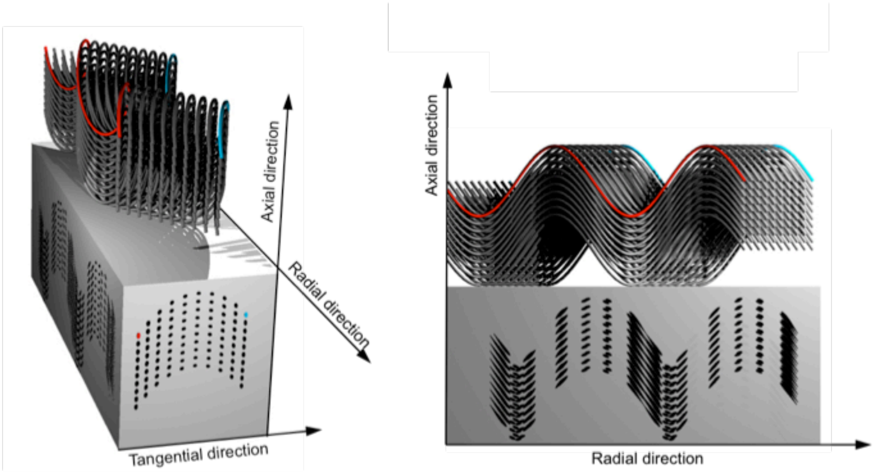

Figure C: 3D model of the distorted helical model showing the continuous phase shift of π after 1 mm in the tangential direction. The continuous phase shift is observed between the red and the turquoise tubule. The arrangement of the tubular cross-sections resulting from a longitudinal and tangential cut is also shown below the helical tubules.
