# Supplementary material for: Relation between the Macroscopic Pattern of Elephant Ivory and Its Three-Dimensional Micro-Tubular Network
Source: PLoS One. 2017 Jan 26;12(1):e0166671. doi: 10.1371/journal.pone.0166671 (PMC5268646; doi:10.1371/journal.pone.0166671)
Supplement: S7 Fig — (PDF) [file pone.0166671.s008.pdf]

S7 Figs. SR-µCT data of the 40 mm^3 volume with 1 µm resolution (voxel-size).

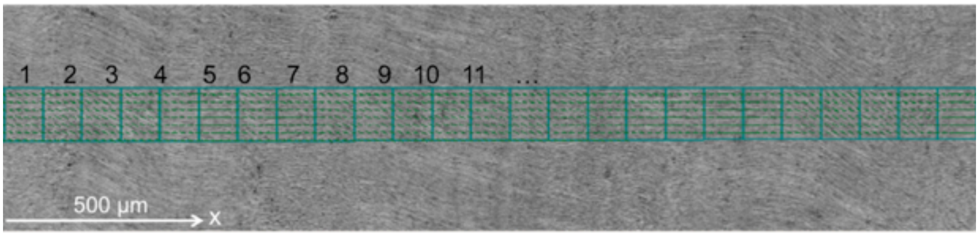

Figure A: 2D virtual section of the longitudinal plane where a sinusoidal trend for the tubule is observed, green squares indicate the segmentation of the 3D volume into 25 ROIs.

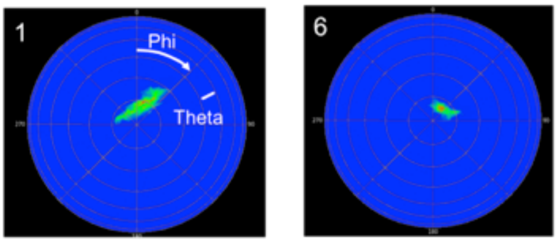

Figure B: determination of the average orientation of the main tubule axis for ROI number 1 and 6, Phi and Theta are represented for ROI 1 and 6 and measured for each ROI.

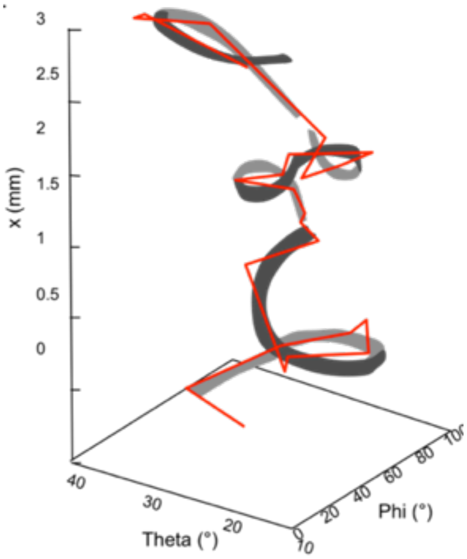

Figure C: 3D plot of Theta and Phi for each ROI, which allows us to evidence a helical shape of tubules.

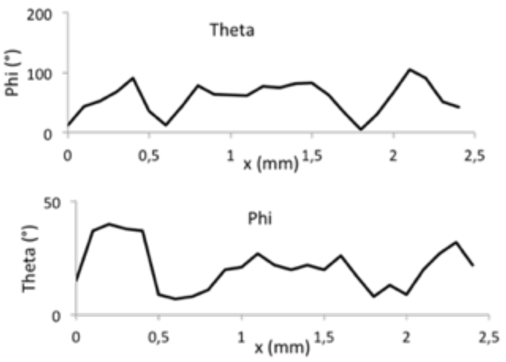

Figure D: 2D plot of Theta (x) and Phi (x) showing the approximated 1 mm pitch of the helix.
