# Supplementary material for: Relation between the Macroscopic Pattern of Elephant Ivory and Its Three-Dimensional Micro-Tubular Network
Source: PLoS One. 2017 Jan 26;12(1):e0166671. doi: 10.1371/journal.pone.0166671 (PMC5268646; doi:10.1371/journal.pone.0166671)

S8 Fig. SR-μCT data of the 5.10^{-4} mm^3 volume with 0.4 μm resolution (voxel-size). 2D SR-μCT images of the three planes, (xy), (xz) and (zy) and 3D visualization of the tubular network in the center.

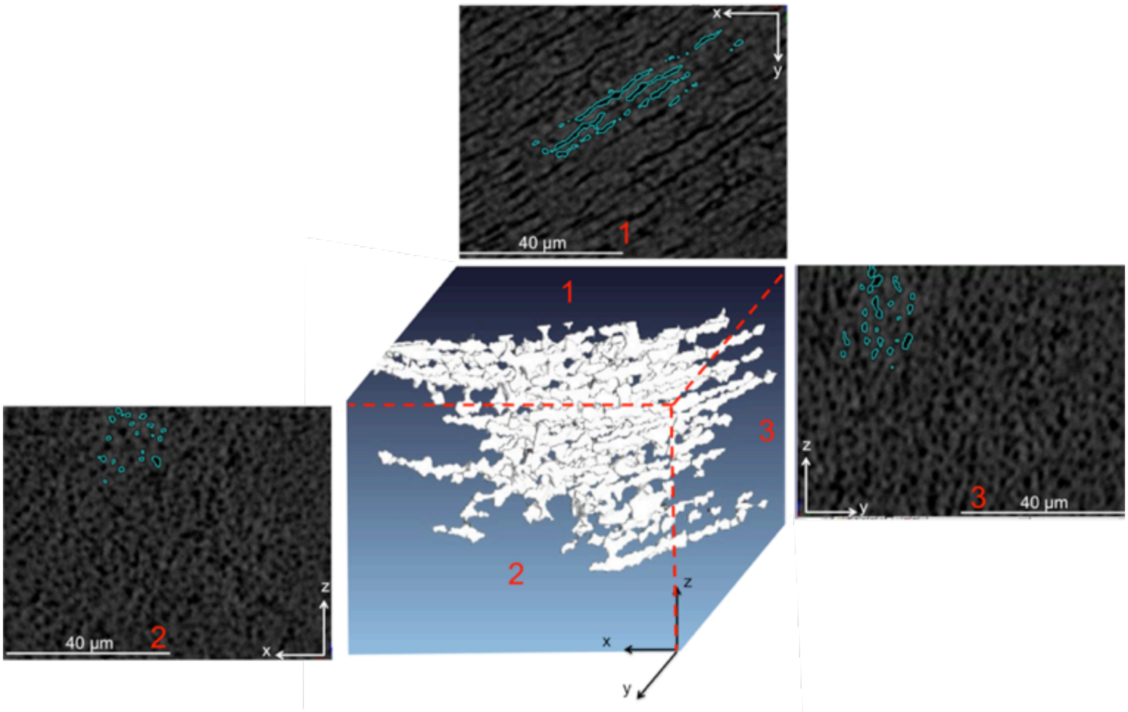

Supplement: S8 Fig — (PDF) [file pone.0166671.s009.pdf]
