# Supplementary material for: Relation between the Macroscopic Pattern of Elephant Ivory and Its Three-Dimensional Micro-Tubular Network
Source: PLoS One. 2017 Jan 26;12(1):e0166671. doi: 10.1371/journal.pone.0166671 (PMC5268646; doi:10.1371/journal.pone.0166671)

S9 Fig. Influence of light on the origin of the Schreger pattern. Two superimposed transverse sections of elephant ivory show the absence of mirror relation between the two sides in contact in terms of the colors of the Schreger pattern.

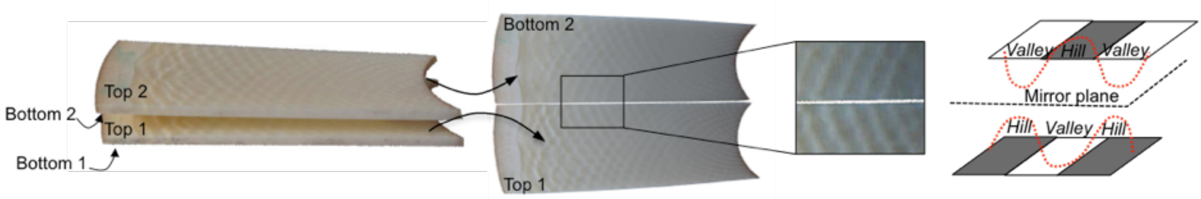

Supplement: S9 Fig — (PDF) [file pone.0166671.s010.pdf]
