# Supplementary material for: Relation between the Macroscopic Pattern of Elephant Ivory and Its Three-Dimensional Micro-Tubular Network
Source: PLoS One. 2017 Jan 26;12(1):e0166671. doi: 10.1371/journal.pone.0166671 (PMC5268646; doi:10.1371/journal.pone.0166671)
Supplement: S1 Text — (PDF) [file pone.0166671.s011.pdf]

S1 Text.

Details on the modeling of the two cubes:

In order to simulate the role of the angle of the cutting plane α with respect to a rotation around an axis perpendicular to the tubules, two simple structures were simulated using the 3D software Rhinoceros (version 4 SR09) with the Grasshopper plugin (version 0-0-0061). The two structures contained linear tubules with a radius of 1 µm, one had a regular (square) array of tubule spaced 6 µm apart as determined from SEM measurements of the tangential plane, the other was an irregular array of tubules using the experimentally measured coordinates of tubules.
