# Supplementary material for: Relation between the Macroscopic Pattern of Elephant Ivory and Its Three-Dimensional Micro-Tubular Network
Source: PLoS One. 2017 Jan 26;12(1):e0166671. doi: 10.1371/journal.pone.0166671 (PMC5268646; doi:10.1371/journal.pone.0166671)
Supplement: S2 Text — (PDF) [file pone.0166671.s012.pdf]

S2 Text. Modeling

In order to calculate the intersection of tubules with the three cutting planes (longitudinal, radial, and tangential) for the different 3D models of the tubular network of ivory, we simulated a variety of different structural models using the 3D software Rhinoceros (version 4 SR09) with the internal “Rhinoscript” scripting language.

The model of Miles and White assumed radially directed sinusoidal tubules (with an amplitude of 250 μm in the axial direction and a period of 1000 μm), spaced 6 μm apart in a square lattice (in the tangential plane). Every 500 μm in the tangential direction, the next group of tubules is phase shifted by an amount of π with respect to the previous group.

The Virag model also assumes sinusoidal tubules (with an amplitude of 300 μm in the axial direction and a period also of 1000 μm), however spaced 13 μm apart in a square lattice in the tangential plane. In contrast to the Miles and White model, this model includes, in addition to the stepwise phase shift of π every 500 μm in the tangential direction, a continuous phase shift in the tubules also in the tangential direction such that each sinusoid is shifted 13 μm in the radial direction with respect to its immediate neighbor in the tangential plane (i.e. a phase shift of 0.013*2π).

The helical model is based on the experimental results of this study, which suggests that the tubules are radially oriented helices that can be described by the equation $(a,t) = (230\sin(\frac{2\pi}{1000}r), 90\cos(\frac{2\pi}{1000}r))$ where a is the axial position of the tubule and t is the transverse position of the tubule with respect to the radially oriented helical axis, and r is the radial position. This model was tested for a regular square lattice of tubules in the tangential plane spaced 6 μm apart. Tubules were simulated 1) without phase shift, 2) with a gradual phase shift of 0.006*2π in the tangential direction and 3) a stepwise phase shift of π every 500 μm and a gradual phase shift of 0.006*2π in the tangential direction.

S10 Fig. highlights how a virtual cut through an array of helical (or sinusoidal) tubules can be obtained through a series of successive cuts through individual tubules, allowing for better memory usage in the computer. Simulation of the intersection of many tubules with a cutting plane (i and vi), which is computationally expensive to do in one step for biologically relevant tubule arrays, we calculated the cross-sections in an iterative manner. The intersection of each individual tubule was calculated (ii-iii), and added to the intersection of the next tubule which is translated a known distance from the previous one (iv-v).
