# Supplementary material for: Relation between the Macroscopic Pattern of Elephant Ivory and Its Three-Dimensional Micro-Tubular Network
Source: PLoS One. 2017 Jan 26;12(1):e0166671. doi: 10.1371/journal.pone.0166671 (PMC5268646; doi:10.1371/journal.pone.0166671)
Supplement: S3 Text — (PDF) [file pone.0166671.s013.pdf]

S3 Text. Influence of light on the origin of the Schreger pattern.

Tubules are basic objects and have a mirror image whereas the colors of the Schreger pattern do not. The figure shows that when the adjacent faces of two serial transverse sections are compared (i.e. the top face of section 1 (Top 1) and the bottom face of section 2 (Bottom 2)), they are not mirror images of one another; rather, the bright bands of one section correspond to the dark bands of the other. We have demonstrated that lines and dots in transverse sections tend to be associated with bright and dark regions, respectively. However, here, hills and valleys of tubules, which are the same shape in transverse cross-section, are associated with different colors of the Schreger pattern in mirror image serial sections. This counterintuitive result was also implicitly shown in the model of Miles and White (1960), who drew a 3D scheme of a transverse ivory section, representing the radial staggering of the bright and dark rhomboids as corresponding respectively to the valleys and hills of sinusoidal tubules.
