# Supplementary material for: Relation between the Macroscopic Pattern of Elephant Ivory and Its Three-Dimensional Micro-Tubular Network
Source: PLoS One. 2017 Jan 26;12(1):e0166671. doi: 10.1371/journal.pone.0166671 (PMC5268646; doi:10.1371/journal.pone.0166671)
Supplement: S4 Text — (PDF) [file pone.0166671.s014.pdf]

S4 Text. Detailed description of the 2D slices obtained by virtually cutting the 3D models

The transverse plane. The experimental transverse plane shows the structural staggered pattern with squared regions of dots and squared regions of lines of ~500 x 500 μm^2. Therefore, line areas (and dot areas) are observed every 1 mm in the radial direction and in the tangential direction (Fig 8a, transverse plane). Moreover, lines of maximum ~200 µm length are observed.

The simulated transverse plane of Miles&White model shows mainly dot features which progressively elongate to ~8 µm length in the radial direction (Fig 8b, transverse plane). No change is observed in the tangential direction. The 8 µm-long patterns occupy regions of ~120 µm size every 500 µm in the radial direction. By introducing a phase shift of $\pi/2$ instead of the one of π suggested by Miles&White model, an additional staggering in the tangential direction is created. Moreover, this modified version of Miles&White model shows longer lines up to ~30 µm.

The simulated transverse plane of Virag model shows also mainly dots (Fig 8b, transverse plane). They progressively elongate to maximum ~25 µm length in the radial direction. A continuous shift of the pattern is observed in the tangential direction and is due to the continuous phase shift of tubules. Diagonal line areas of ~100 µm size are present every ~500 µm.

The simulated transverse plane of the helical model without phase shift of the tubules shows alternated dot and line regions in the radial direction every 500 µm. Individual lines are ~30 µm and occupy regions of ~250 µm size (Fig 9a, transverse plane).

The simulated transverse plane of the helical model with a continuous phase shift of π after 1 mm shows diagonal areas of ~375 µm size every 750 µm containing lines of 45 µm length. Overlaps of helical tubules are observed every 750 µm (Fig 9b, transverse plane).

The simulated transverse plane of the helical model with a continuous phase shift of π after 1 mm and a stepwise phase shift of π after 0.5 mm shows too many overlaps to be considered for representing the experimental data (Fig 9c, transverse plane).

The longitudinal plane. The experimental longitudinal plane shows alternating vertical (in the axial direction) bands (~500 µm wide) of 100 µm-long line regions (lines inclined ~45° to the vertical of the section) and shorter line areas. Therefore, line regions (and dot regions) are observed every 1 mm in the radial direction. The juxtaposition of the long and short line regions present a sinusoidal trend in the radial direction (wavelength of 1 mm) (Fig 8a, longitudinal plane).

The simulated longitudinal planes of Miles&White and Virag models show complete sinusoids with a wavelength of 1 mm in the radial direction (Fig 8b 8c, longitudinal plane).

The arrangement of the tubular sections of the simulated longitudinal plane of the helical model without phase shift changes in the radial direction and stays the same in the tangential one (Fig 9a, longitudinal plane). It shows regions of dots and lines every ~300 µm with lines of 10 µm length.

The simulated longitudinal plane of the helical model with a continuous phase shift of π after 1 mm shows a very similar pattern to the previous model, with regions of dots and lines observed every ~750 µm. Vertical overlaps in the axial direction are present every 1 mm (Fig 10b, longitudinal plane).

The simulated transverse plane of the helical model with a continuous phase shift of π after 1 mm and a stepwise phase shift of π after 0.5 mm shows the same pattern as the previous model (Fig 10c, transverse plane).

The tangential plane. The experimental tangential plane shows a periodic change of orientation of the ellipsoidal cross-sections every 1 mm (Fig 8a, tangential plane).
The simulated tangential plane of Miles&White presents a regular array of circular tubular cross-sections. Modified Miles&White model shows in the tangential direction alternating circular and ellipsoid cross-section areas. Circular (and ellipsoid) areas are observed every 1 mm (Fig 8b, tangential plane).
The simulated tangential plane of Virag model shows periodic changes in the size of the cross-sections of the tubules in the tangential direction every ~500 µm (Fig 8b 8c, tangential plane), not every 1 mm as it was suggested in Virag (2012) (S6 Fig.).
The helical model without phase shift shows an array of regularly spaced ellipsoids and with the same orientation (Fig 9a, tangential plane).
The helical model with the continuous phase shift of π after 1 mm shows the periodic change of orientation of the ellipsoidal cross-sections every 1 mm. However, periodic overlaps every 1 mm are present in the simulated plane (Fig 9b, tangential plane).
The simulated tangential plane of the helical model with a continuous phase shift of π after 1 mm and a stepwise phase shift of π after 0.5 mm shows too many overlaps every 500 µm (Fig 9c, tangential plane).
